# Acute Respiratory Failure Secondary to COVID-19 Viral Pneumonia Managed With Hydroxychloroquine/Azithromycin Treatment

**DOI:** 10.7759/cureus.8268

**Published:** 2020-05-25

**Authors:** Kenneth K Ng, Mitchell K Ng, Angelina Zhyvotovska, Sahib Singh, Ketan Shevde

**Affiliations:** 1 Anesthesiology, State University of New York Downstate Medical Center, Brooklyn, USA; 2 Orthopaedic Surgery, Maimonides Medical Center, Brooklyn, USA; 3 Internal Medicine, State University of New York Downstate Medical Center, Brooklyn, USA; 4 Neuroscience, Johns Hopkins University, Baltimore, USA

**Keywords:** covid-19, acute respiratory failure, viral pneumonia, hydroxychloroquine, azithromycin

## Abstract

A 74-year-old male was admitted to the Intensive Care Unit (ICU) at State University of New York (SUNY) Downstate Medical Center following acute respiratory failure secondary to coronavirus disease 2019 (COVID-19) viral pneumonia. The patient had significant comorbidities, including a history of lung and esophageal cancer status-post resection, cerebrovascular accident with neurological deficits, diabetes mellitus, hypertension, and peripheral vascular disease. The patient was in septic shock and respiratory failure on admission requiring intubation and mechanical ventilation. Computed tomography (CT) of the chest showed patchy bilateral opacities suspicious for viral pneumonia and the COVID-19 sputum sample sent to the New York Department of Health returned positive. This patient’s comorbidities, along with his age, placed him in the highest risk of mortality for COVID-19. The patient was managed pharmacologically with hydroxychloroquine and azithromycin. By Day 5 of his admission, he improved significantly and was extubated and downgraded from the ICU to the medical floor, pending discharge. This case report provides anecdotal evidence for the effectiveness of the hydroxychloroquine and azithromycin combination currently being used across the nation to manage COVID-19, pending development of a definitive vaccine or antiviral treatment

## Introduction

The severe acute respiratory syndrome caused by coronavirus 2 (SARS-CoV-2) is the cause of a global pandemic that began in 2019 with significant mortality for patients worldwide [1]. The coronavirus disease of 2019, abbreviated as COVID-19 by the World Health Organization (WHO), has relatively high mortality and transmissibility for a viral disease [2]. The official fatality rates of 0.66% in the United States (US) as per the Centers for Disease Control and Prevention (CDC) and 2.3% as per Chinese data are probably overestimated, given the number of asymptomatic carriers, but there is no doubt that COVID-19 has overwhelmed healthcare systems globally [3]. As of March 2020, there was no definitive vaccine or antiviral treatment despite ongoing efforts to develop these.

SARS-COV-2 causes acute respiratory distress syndrome (ARDS) in those who are elderly or who have significant cardiopulmonary comorbidities [1]. A recent published small clinical trial in France has shown that combination pharmacological treatment with hydroxychloroquine, an old antimalarial drug, and azithromycin, an antibiotic, led to decreased viral load and an average carrying duration in COVID-19 patients [4]. The French study was inspired by a smaller Chinese study showing similar improvements [5]. These studies have led many healthcare providers globally to utilize hydroxychloroquine/azithromycin combinations to treat COVID-19 patients in critical care settings [6].

The State University of New York (SUNY) Downstate Medical Center in Brooklyn, New York is designated as a COVID-only patient hospital, as per New York Governor Cuomo’s order on March 28, 2020. COVID-19 patients at SUNY Downstate receive a combination of hydroxychloroquine and azithromycin. This case report examines the clinical course of one of the first patients to receive the treatment, an elderly African American male with ARDS secondary to COVID-19. Despite significant comorbidities, the patient steadily improved throughout his intensive care unit (ICU) stay and was extubated by the fifth day of his admission. He was transferred to the medical floor for continued treatment for community-acquired pneumonia (CAP). This case, while anecdotal, supports the efficacy of hydroxychloroquine and azithromycin as a safe and effective treatment for COVID-19.

## Case presentation

The patient is a 74-year-old male living in a nursing home in New York that was a known focus of a COVID-19 outbreak. He has a past medical history significant for squamous cell carcinoma of the lung with the invasion of the esophagus status-post resection and chemoradiation seven years earlier, a cerebrovascular accident with right residual weakness, diabetes mellitus, hypertension, and peripheral vascular disease (PVD). He was brought to SUNY Downstate Medical Center in septic shock with altered mental status, fever, tachycardia, and hypotension. The patient’s renal function was preserved with a baseline creatinine of 0.7 mg/dL. On presentation, he was in acute respiratory failure with desaturation to 70% while on 6 liters/minute of nasal oxygen. He required intubation and mechanical ventilation.

Upon transfer to the ICU, the patient underwent organ system-based management for sepsis and ARDS secondary to COVID-19 viral pneumonia complicated by a secondary bacterial infection. He was maintained on mechanical ventilation on volume control mode and sedated with dexmedetomidine drip. During the first two days of admission, he required vasopressor support with norepinephrine. On the third day, norepinephrine was titrated off and he remained hemodynamically stable without vasopressor support. He had an episode of acute kidney injury (AKI) which resolved with the initiation of free water flush of 200 mL every six hours and 125 mL per hour of intravenous (IV) normal saline, which was later stopped. His chest x-ray on admission showed bilateral lung opacities and focal right lower lobe consolidation consistent with viral pneumonia complicated by a bacterial superinfection. COVID-19 test on patient’s sputum performed by the New York Department of Health was positive. Blood cultures grew methicillin-sensitive *Staphylococcus aureus* (MSSA).

As per recommendations of the infectious disease (ID) team, the patient was one of the first at SUNY Downstate to receive pharmacological treatment for COVID-19 consisting of hydroxychloroquine, 400 milligrams (mg) twice a day for one day, followed by 200 mg twice a day for four days, in addition to azithromycin, 500 mg once the first day and 250 mg for four days. This was based on a recently published study from France which demonstrated combination treatment with hydroxychloroquine and azithromycin led to improved outcomes in COVID-19 patients measured by decreased viral count taken from nasal swabs [4]. The patient was also started on IV vancomycin when blood cultures came back positive for MSSA infection.

**Figure 1:**
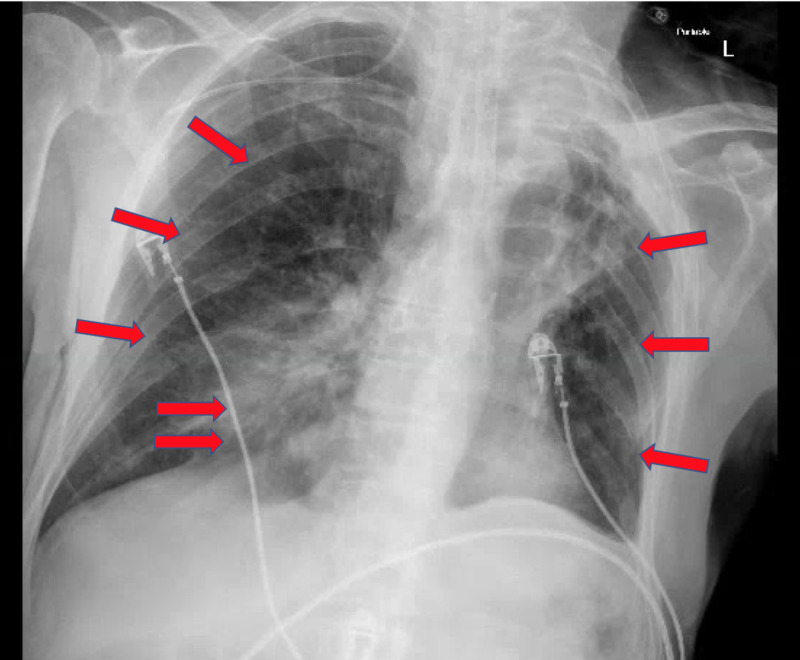
Initial chest x-ray on Day 1 of hospital admission shows bilateral patchy opacities secondary to severe acute respiratory syndrome coronavirus 2 (SARS-CoV-2) viral pneumonia and right lower lobe consolidation secondary to bacterial superinfection

**Figure 2:**
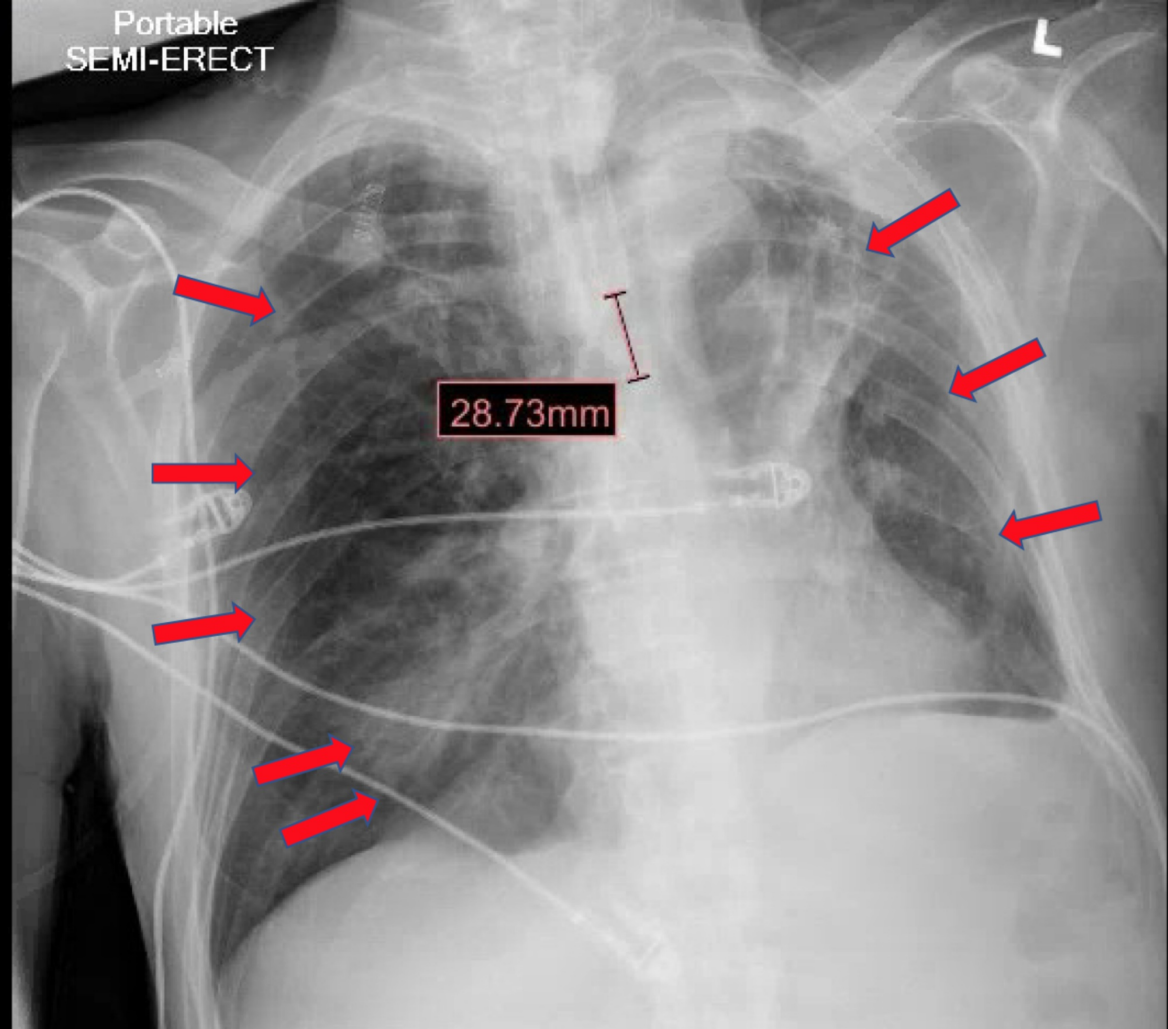
Chest x-ray on Day 5 of hospital course, status-post hydroxychloroquine and azithromycin treatment and just prior to extubation and downgrade from the intensive care unit (ICU), showing improvement of bilateral patchy opacities (arrows) and decreased right lower lobe consolidation (arrows)

**Figure 3:**
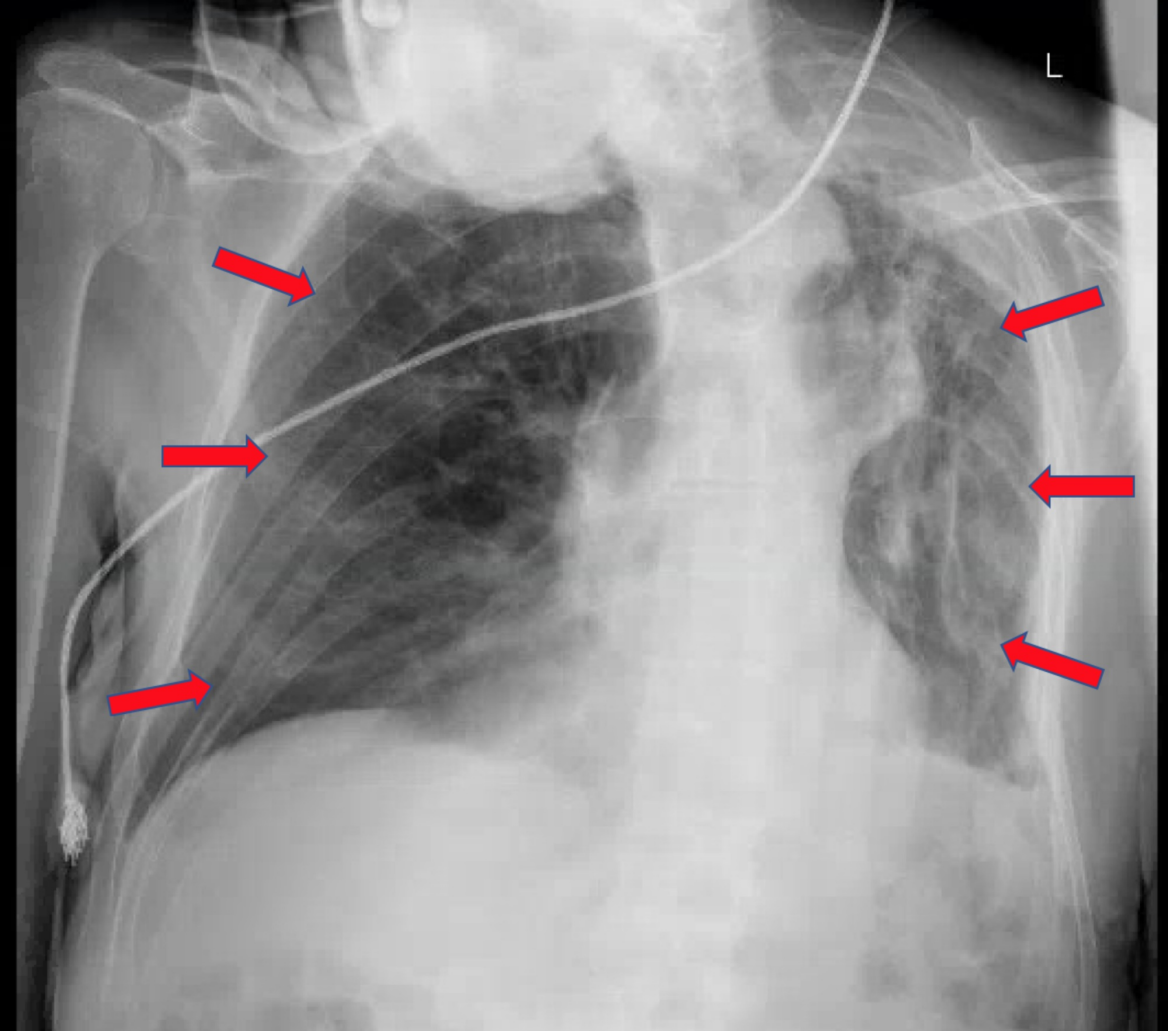
Chest x-ray on Day 9 of hospital course, after transfer from the intensive care unit (ICU) to the COVID-only medical floor, showing continued decrease of lung opacities bilaterally (arrows) with improved visualization and near resolution of right lower lobe consolidation (arrows)

Despite his poor prognosis on admission, the patient steadily improved. Daily chest x-rays showed a gradual resolution of the bilateral patchy infiltrates and right lower lobe consolidation (Figures 1-3). 

The patient remained hemodynamically stable with adequate urine output and without the use of vasopressors. The patient’s mental status improved, and by Day 3, he responded to voice and command at the bedside. Serial arterial blood gases showed improvement in oxygenation even as ventilator settings were gradually decreased with decreasing fraction of inspired oxygen (FiO_2_), positive expiratory end pressure (PEEP), and respiratory rate. The patient passed a continuous positive pressure (CPAP) spontaneous breathing trial on Day 5 of admission with a rapid shallow breathing index (RSBI) < 100 and was successfully extubated. He was downgraded to a COVID-only medical floor with appropriate droplet and contact isolation precautions pending discharge.

## Discussion

This case report documents the hospital course of a critically ill COVID-19 patient who improved with critical care in the ICU on a five-day regimen of hydroxychloroquine and azithromycin. It provides a demonstration of a highly positive outcome in a patient at high risk for mortality, given his advanced age and numerous comorbidities. The mortality rate for patients in this age group is 8.0%, and given the patient’s numerous comorbidities, his risk was likely higher [7-8]. The patient’s relatively transient need for mechanical ventilation (totaling five days), rapid improvement in mental status, rapid resolution of septic shock, and resolution of his infection of radiological imaging support the potential efficacy of hydroxychloroquine and azithromycin as a combination treatment for COVID-19. While this case report is not a substitute for a randomized clinical trial to determine the efficacy of hydroxychloroquine/azithromycin, this patient’s hospital course is representative of a case series of least 11 other patients at SUNY Downstate who received the hydroxychloroquine and azithromycin and recorded a positive outcome, either downgrade from the ICU or discharge from the hospital.

**Figure 4:**
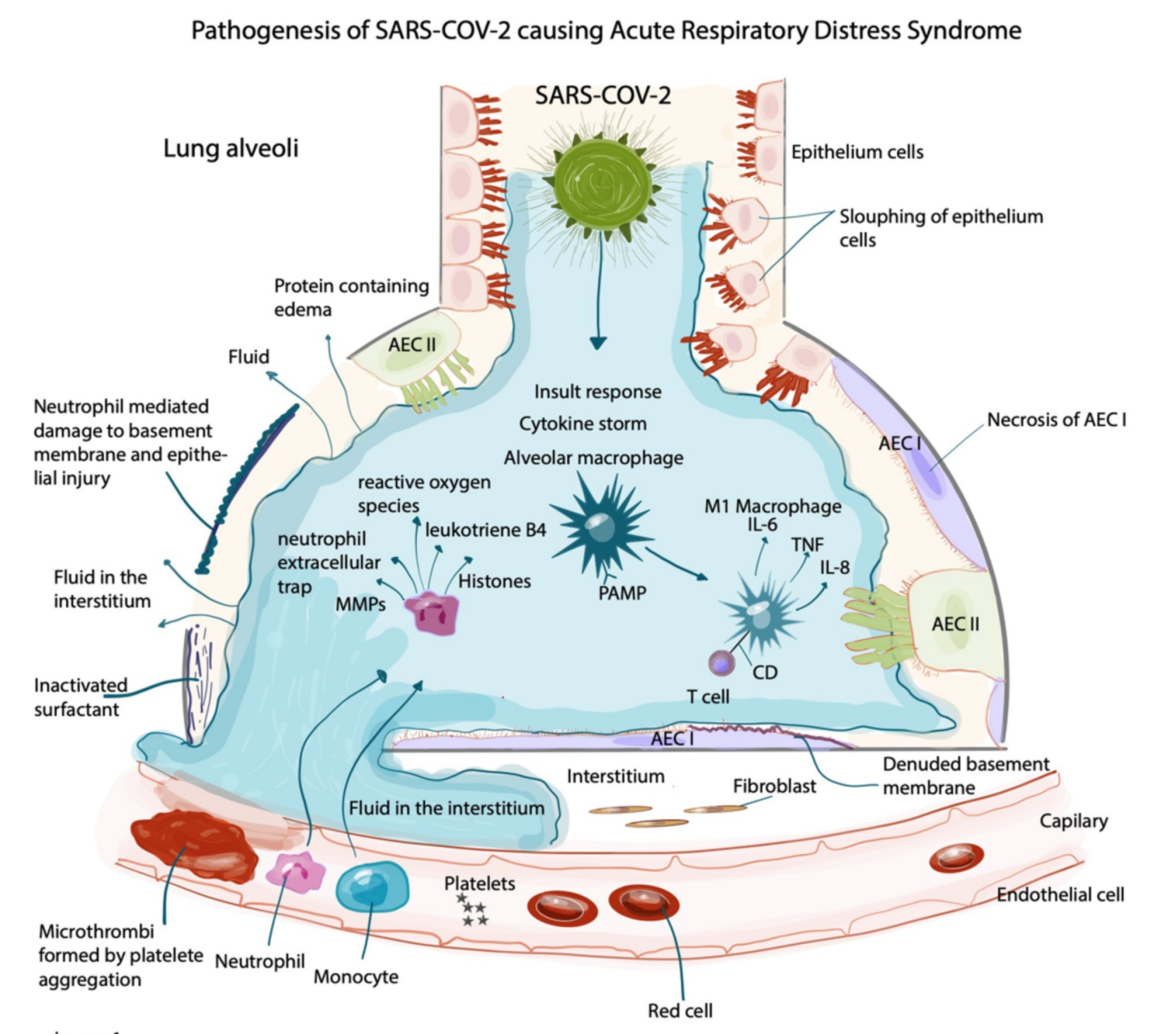
Pathophysiology of SARS-CoV-2 causing acute respiratory distress syndrome AEC I: type 1 alveolar epithelial cell; AEC II: type 2 alveolar epithelial cell; IL 6: interleukin 6; IL 8: interleukin 8; MMPS: matrix metalloproteinase; PAMP: pathogen-associated molecular pattern; SARS-CoV-2: severe acute respiratory syndrome coronavirus 2; TNF: tumor necrosis factor

Though the mechanism is not well-understood, it is hypothesized that hydroxychloroquine and azithromycin may work in several ways. First, hydroxychloroquine and chloroquine reduce the glycosylation of angiotensin-converting enzyme 2 (ACE2) receptors, which the SARS-CoV-2 use to enter host cells (Figure 4) [7]. 

Second, it is hypothesized that hydroxychloroquine decreases the production of pro-inflammatory cytokines whose expression lead to the ARDS pathway [8]. Finally, large amounts of the virus are known to enter host cells through endocytosis. Chloroquine accumulates in endosomes, decreasing the space available for viruses like SARS-CoV-2, and also raises endosomal pH, interfering with the entry and exit of viruses from host cells [9]. Although a potential mechanism for azithromycin is not well-defined, it is noted that in the oft-cited French study showing decreased COVID viral loads, 70% of patients in the group who received hydroxychloroquine alone showed no viral load by Day 6, while 100% of patients in the group who received hydroxychloroquine-azithromycin showed no viral load by Day 6 (versus 12.5% patients who received no treatment at all), pointing to a synergistic effect with hydroxychloroquine [4].

COVID-19 is a novel viral respiratory illness that has caused a large pandemic with significant mortality in 2020 [10]. As of March 2020, there is no curative treatment for COVID-19, with the first antiviral agents and vaccines anticipated to appear in the fall of 2020. To date, the management of critically ill COVID-19 patients is highly provider and institution-dependent. In many hospitals, including SUNY Downstate where this case was documented, the most common drug combination used is hydroxychloroquine and azithromycin. This case study and the patient’s relatively rapid recovery adds to the body of evidence supporting the potential use of hydroxychloroquine and azithromycin as empiric treatments for COVID. The safety of both hydroxychloroquine and azithromycin is not likely a significant issue, given both drugs have been safely utilized for decades for malaria prophylaxis and to treat community-acquired pneumonia, respectively [11]. 

## Conclusions

In summary, this case report highlights the course and recovery of an elderly COVID-19 patient with many comorbidities typically associated with a high rate of mortality. This case supports the need for further study on the topic of appropriate management. A larger randomized clinical trial assessing the efficacy of a combination treatment hydroxychloroquine and azithromycin on COVID-19 patients should be conducted.
